# Impacts of a New Supermarket on Dietary Behavior and the Local Foodscape in Kisumu, Kenya: Protocol for a Mixed Methods, Natural Experimental Study

**DOI:** 10.2196/17814

**Published:** 2020-12-21

**Authors:** Louise Foley, Oliver Francis, Rosemary Musuva, Ebele RI Mogo, Eleanor Turner-Moss, Pamela Wadende, Vincent Were, Charles Obonyo

**Affiliations:** 1 MRC Epidemiology Unit University of Cambridge Cambridge United Kingdom; 2 Centre for Global Health Research Kenya Medical Research Institute Kisumu Kenya; 3 Faculty of Education and Human Resources Kisii University Kisii Kenya

**Keywords:** food retail, food environment, supermarket, natural experiment, diet, Africa

## Abstract

**Background:**

Access to healthy food is considered a key determinant of dietary behavior, and there is mixed evidence that living near a supermarket is associated with a healthier diet. In Africa, supermarkets may contribute to the nutrition transition by offering both healthy and unhealthy foods and by replacing traditional food sellers. In Kisumu, Kenya, a planned hypermarket (ie, a supermarket combined with a department store) will form the basis for a natural experimental evaluation.

**Objective:**

The aim of this study is to explore the impacts of a new hypermarket on food shopping practices, dietary behaviors, physical activity patterns, and body composition among local residents and to identify concurrent changes in the local foodscape. We also aim to explore how impacts and associations vary by socioeconomic status.

**Methods:**

We employ a mixed methods, longitudinal study design. Two study areas were defined: the hypermarket intervention area (ie, Kisumu) and a comparison area with no hypermarket (ie, Homabay). The study is comprised of 4 pieces of primary data collection: a quantitative household survey with local residents, a qualitative study consisting of focus group discussions with local residents and semistructured interviews with government and private sector stakeholders, an audit of the local foodscape using on-the-ground data collection, and an intercept survey of shoppers in the hypermarket. Assessments will be undertaken at baseline and approximately 1 year after the hypermarket opens.

**Results:**

Baseline assessments were conducted from March 2019 to June 2019. From a total sampling frame of 400 households, we recruited 376 of these households, giving an overall response rate of 94.0%. The household survey was completed by 516 individuals within these households. Across the two study areas, 8 focus groups and 44 stakeholder interviews were conducted, and 1920 food outlets were geocoded.

**Conclusions:**

This study aims to further the understanding of the relationship between food retail and dietary behaviors in Kenya. Baseline assessments for the study have been completed.

**International Registered Report Identifier (IRRID):**

DERR1-10.2196/17814

## Introduction

Dietary behavior is complex and shaped by multiple interacting factors, including policy, built, and social environments [1]. Access to healthy food is considered a key determinant of dietary behavior, and some evidence suggests, albeit inconsistently, that residential proximity to a supermarket is associated with a healthier diet [2]. In high-income countries, there is mixed evidence that the establishment of a new supermarket influences health-related outcomes among local residents [3-5]. A review indicated inconsistent evidence that new supermarkets improve fruit and vegetable consumption and no evidence for changes in BMI or self-rated health [3]. However, further reviews of research from predominantly high-income countries suggested that interventions that involved manipulating the price or availability of products within grocery stores could increase the purchasing of healthier foods [6,7].

Evidence on the relationship between supermarkets, food purchasing, diet, and health is drawn mostly from high-income countries. There is an inadequate understanding of dietary behaviors and their drivers in low- and middle-income countries (LMICs). It is particularly important to understand dietary behaviors in these settings because of the ongoing nutrition transition that is typified by a movement away from traditional staples toward the consumption of cheap and highly processed food [8]. This is responsible, in part, for the emerging “triple burden of malnutrition,” whereby undernutrition and micronutrient deficiency coexist with obesity at population, household, and individual levels [9]. In Africa, the structure of the food retail market is changing rapidly as large supermarket chains expand into urban settings [10]. Supermarkets may play a key role in driving the nutrition transition through increasing the availability of processed food, though they may also sell healthy food cheaply or facilitate access to a wider range of foods [10,11]. Due to economies of scale, supermarkets are often able to sell items more cheaply than local traders and have been shown to displace smaller or informal local food outlets frequented by low–socioeconomic status (SES) households, generating or entrenching inequalities in food access and health [10].

Kenya is a low-income [12] East African country with a population of approximately 50 million [13]. Kenya is experiencing an escalating burden of noncommunicable disease and obesity [14,15], which is related, in part, to changing local diets. Kenya has a prospering supermarket sector [11], accounting for approximately 10% of the share of grocery sales at the national level, with a higher share in urban centers [16]. Evidence from Kenya suggests that living near, or shopping at, a supermarket increased the consumption of processed food at the expense of unprocessed food [11,16] and was associated with a higher BMI and metabolic syndrome in adults [16-18] but a lower probability of being underweight in children [17].

**Figure 1:**
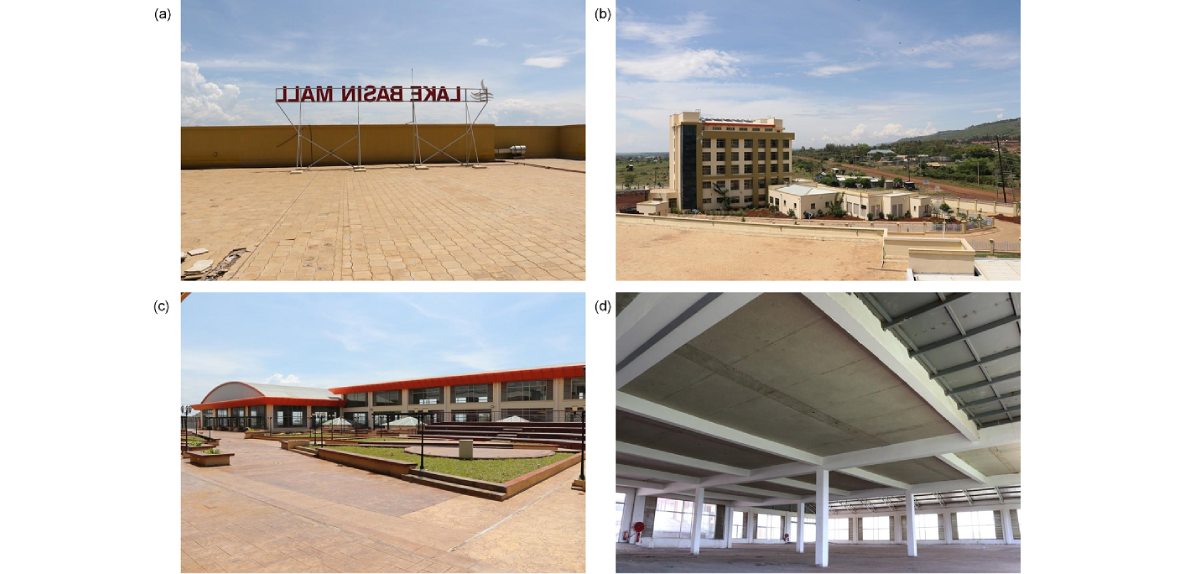
Images showing (a and b) the Lake Basin Mall site, (c) a scene from within the mall, and (d) the hypermarket site.

A new hypermarket (ie, a supermarket combined with a department store) is currently under construction at Lake Basin Mall in Kisumu, Western Kenya (see Figure 1). The mall, funded through a public-private partnership and currently planned to be the largest in Western Kenya, is located approximately 5 km from the central business district of the city of Kisumu, the seat of Kisumu County. The mall will house other amenities, such as an amphitheater, doctors’ offices, cafeterias, a gym, and office spaces. Lake Basin Mall is in close proximity to a higher-SES residential area but also close to lower-SES informal (ie, slum) settlements. It is situated along a busy highway that opens up the western part of Kenya, runs past Kisumu International Airport, and continues on toward Uganda’s Northern Transport Corridor, a popular destination for Kenyan traders in commodities such as fish, fruit, cereals, and animal feed.

The opening of the hypermarket provides an opportunity to examine potential impacts on dietary behaviors among residents of Kisumu living nearby and on the local foodscape, using a natural experimental design [19], as well as the opportunity to explore whether impacts are distributed unequally or inequitably. Impacts will be examined relative to the town of Homabay in Homabay County, a neighboring comparison area where no hypermarket is planned. As such, the overall purpose of this study is to address the following research questions:

1. What are the individual, household, and population impacts of the new hypermarket at Lake Basin Mall on food shopping practices, dietary behaviors, physical activity patterns, and body composition among local residents across different SES groups?
2. How has the local foodscape changed, do these changes relate to the new hypermarket at Lake Basin Mall, and how are they experienced by local residents?

## Methods

### Design

This is a protocol for a mixed methods, natural experimental study [20] that will use a combination of quantitative and qualitative research methods to evaluate changes in dietary and health behavior coupled with furthering an understanding of the drivers and impacts of these changes. As is typical for natural experimental studies, the intervention will not be under the control of researchers, and intervention assignment will not be randomized or blinded.

The study is comprised of four core pieces of primary data collection:

1. A longitudinal quantitative household survey with local residents.
2. A longitudinal qualitative study consisting of focus group discussions with local residents and semistructured interviews with government and private sector stakeholders.
3. A longitudinal audit of the local foodscape using on-the-ground data collection.
4. A cross-sectional intercept survey of shoppers in the hypermarket.

The baseline data collection took place in 2019, before the anticipated opening of the hypermarket. The follow-up data collection is anticipated to take place in 2021, approximately 1 year after the hypermarket opens, which should provide sufficient time for shopping habits to adjust after an initial period where use of the hypermarket may be atypical. Assessments will ideally be seasonally matched (ie, they will take place at a similar time at both the intervention and comparison sites) to account for seasonal variation in behaviors. Should this not happen, seasonality will be addressed in the analysis. Modest incentives will be used to increase retention and to compensate for time spent participating in the study. The incentives include transport reimbursement, refreshments, and bars of soap as tokens of appreciation. If households subsequently move to outside the study areas, they will still be eligible for the follow-up assessment. Depending on attrition, we will also consider the possibility of recruiting a new sample of households from the study areas at follow-up.

### Setting

The study is set in Kisumu County and Homabay County in Western Kenya. Kisumu County has a population of 968,909, and Homabay County has a population of 963,794. Two study areas were defined: the hypermarket intervention area (ie, Mamboleo, Kisumu) and a comparison area with no hypermarket (ie, Sofia, Homabay). These areas were delineated using existing spatial census data, field visits, and local knowledge of the study investigators. A 2-km radial buffer was drawn around the hypermarket and matched according to population density with a 2-km radial buffer around another landmark in the comparison area. Therefore, both study areas are 2 km in size, by radius, and the two areas lie approximately 100 km apart.

Though we aimed to ensure similar aggregate socioeconomic characteristics and broadly similar topographical and food retail characteristics apart from the presence of a hypermarket, the sites are still somewhat heterogeneous. The intervention site may be considered urban and the comparison site maybe be considered periurban. However, both areas include a mix of lower- and higher-SES areas, both informal and formal settlements; both areas have a similar number of supermarkets and encompass the main shopping street; and both areas have major roads. Both areas are located on the shores of Lake Victoria, a freshwater lake and the traditional ancestral lands for the Luo ethnic community of Kenya; thus, they share similar cultural practices.

### Eligibility

#### Household Survey

Households living in one of the local study areas at baseline are eligible for the household survey. Individuals are considered household members if they regularly share meals, live together, and pool their monetary resources. A maximum of 5 adults, aged 18 years or over, in the household, including the head of household and the person who is usually responsible for food purchasing, will be eligible to complete interviewer-administered questionnaires and anthropometric measurements. The household head is the adult member of the household who is accepted and recognized by the other household members as the head, which implies a prominent role in making decisions that concern the household. Additionally, a maximum of 5 children, aged between 5 and 17 years, in the household will be eligible to complete anthropometric measurements. Households where either the head or the person responsible for food purchasing is under the age of 18 years will be excluded from the study.

#### Focus Groups

Adult householders from each of the local study areas who took part in the household survey will be eligible to participate in subsequent focus group discussions. These will be disaggregated into male and female as well as lower- and higher-SES groups. This was considered necessary to address any social, economic, cultural, or class issues that may impede free discussion. The focus group discussions will be conducted in local community halls owned by churches, local businesses, or the county government.

#### Stakeholder Interviews

Eligible stakeholders of interest include county government officers from the health, trade, agriculture, and infrastructure departments. Local administration representatives include the county commissioner and representatives of this office. Members of the Lake Basin Development Authority who developed the mall and hypermarket as well as representatives from the local food retail sector, such as the market master, are also considered important stakeholders. Other important local representatives include the motorcycle taxi (ie, boda boda) and fishing industries as well as local faith and religious leaders who are influential in the community.

#### Shopper Intercept Survey

Once the hypermarket is open, shoppers will be eligible to answer questions on their shopping habits and will be intercepted while they shop.

### Intervention

The intervention under study is the new hypermarket at Lake Basin Mall and its associated changes to the local foodscape. At the group level, the intervention dose is operationalized as the geographical proximity to the new mall (ie, living in either the intervention or comparison area). At the individual level, the intervention dose is operationalized according to whether the individual shops at the new hypermarket.

### Outcomes

The primary outcomes are as follows:

1. Change in the self-reported percentage share of monthly household food expenditure from supermarkets between baseline and follow-up.
2. Changes in the number and type of local food outlets between baseline and follow-up.

The secondary outcomes are as follows:

1. Change in self-reported, monthly, household food expenditure, in Kenyan shillings, between baseline and follow-up.
2. Change in self-reported household food security between baseline and follow-up.
3. Change in self-reported individual dietary diversity (ie, number of food groups consumed on the previous day) between baseline and follow-up.
4. Change in measured individual body composition (ie, BMI in kg/m^2^) between baseline and follow-up.
5. Change in self-reported individual well-being between baseline and follow-up.
6. An understanding of the changes that have occurred and how their effects have been experienced.

### Sample Size

The primary outcome is the percentage share of the monthly household food expenditure from supermarkets. With reference to a previous study on supermarkets and food purchasing in Kenya [18], a baseline sample of 250 households—125 intervention and 125 comparison households—was estimated to allow the detection, with 95% confidence and 80% power, of a difference between intervention and comparison groups of 5% in expenditure share, while allowing for a 50% attrition rate between baseline and follow-up. Therefore, we aimed to recruit 300 households at baseline in 2019 to ensure adequate statistical power. While we did not anticipate 50% attrition, we used this as an upper limit to ensure we recruited an adequate number of households at baseline.

### Sampling and Recruitment

#### Household Survey

In each of the study areas, we consulted with local community health volunteers. In the Kenyan health system, community health volunteers represent the first basic level of care. The majority of these volunteers have completed 8 years of primary education and are elected or nominated by their communities to serve them in a voluntary capacity on health-related matters under the community health strategy [21]. A community health volunteer typically works in a village with about 100 households and is supervised by community health extension workers, who are government employees [22]. Community health volunteers visit households monthly to collect general health indicators and often work with partners in their respective areas. They are assumed to know the households well in terms of living standards.

We engaged with 20 community health volunteers from the intervention area and 20 from the comparison area. Each community health volunteer provided a household list from their area, for a total of about 2000 households per study area. In addition, community health volunteers classified each household as low, medium, or high SES. These classifications were broadly based on asset-based measures used in previous household surveys conducted in LMICs [23], in particular, focusing on housing characteristics (eg, material of dwelling floor and roof and main cooking fuel) and access to basic services (eg, electricity supply, source of drinking water, and sanitation facilities), modified for the local context.

Using a geographic information system (GIS), we plotted a quadrant from the center of each study area to demarcate the area into four subareas—northeast, northwest, southeast, and southwest— and plotted three radii of 500 m, 1 km, and 2 km from the center. Each household in the sampling frame was classified by quadrant, distance from the center, and SES. A stratified random sample of households was drawn from these three strata—400 in total (200 in the intervention area and 200 in the comparison area)—to account for refusal to participate.

Study teams consisting of two field workers, guided by a local community health volunteer, will knock at the doors of eligible households to make an initial approach and recruit households to participate in the household survey.

#### Focus Groups

Following completion of the household survey, a purposive sample of participants will be drawn for the resident focus groups. We will aim for a diverse sample that mirrors the basic demographic characteristics of the survey sample and includes a mixture of lower- and higher-SES participants, those living in formal and informal settlements, both males and females, and a range of ages. Householders will be approached and recruited by the same field worker teams using contact details provided during the household survey. We aim to conduct four focus groups in each study area, split by gender and SES.

#### Stakeholder Interviews

We will use existing relationships combined with snowball sampling to identify and recruit a range of stakeholders to participate in semistructured interviews, including mall developers and managers as well as members of the county government, including the trade executive, the health executive, the planning executive, and the local ward representative. We will give preference to those directly involved in the decision making and development of the mall and aim to interview at least 6 stakeholders per study area.

#### Shopper Intercept Survey

Once the hypermarket is open, a minimum of 20 shoppers will be recruited while they shop to answer a short survey.

### Assessment

#### Household Survey

At baseline and follow-up, participants will complete a household and an individual questionnaire. The household questionnaire will be completed by the head of household and by the person who is usually responsible for food purchasing and will assess the following variables:

1. Demographic variables for each individual in the household (ie, age, sex, relationship to head of household [eg, wife or child], ethnicity, education, and occupation).
2. Characteristics of the household (ie, housing tenure and quality, access to electricity and water, appliance used for cooking, refrigerator ownership, car ownership, and duration of residence in the area).
3. Household food purchasing in the previous month, including sources of food, types of food retail accessed, and amount of money spent at different types of food retailers and overall.
4. Number of trips made to purchase food and modes of transport used in the previous month.
5. Household food security, based on the Food and Agriculture Organization’s Food Insecurity Experience Scale [24].

The individual questionnaire will be completed by each eligible adult, aged 18 years or over, and will assess the following variables:

1. Dietary diversity using an unquantified 24-hour dietary recall and following the approach described by the Food and Agriculture Organization and the United States Agency for International Development’s Food and Nutrition Technical Assistance III Project [25]. In order to calculate a dietary diversity score, the range of foods and drinks consumed will be classified into 10 core food groups: grains, white roots, tubers, and plantains; pulses; nuts and seeds; dairy; meat, poultry, and fish; eggs; dark green leafy vegetables; other vitamin A–rich fruit and vegetables; other vegetables; and other fruit.
2. Physical activity, using the World Health Organization Global Physical Activity Questionnaire [26].
3. Well-being, using two items from the abbreviated version of the World Health Organization Quality of Life instrument [27].
4. Social connectedness, using 3 items from the Social Provisions Scale [28].

Following this, all eligible participants in the household, both adults and children, will complete anthropometric measurements (ie, height, weight, and waist circumference) using standard procedures. A tape measure, stadiometer, and calibrated scales will be used. Each measurement will be taken in duplicate.

Assessments will last approximately two hours and will be conducted on different days of the week in order to capture both weekdays and weekends on the dietary recall. Although, ideally, multiple dietary recalls would be used, in order to minimize participant burden we will undertake a single dietary recall to describe the range of food groups consumed on the recall day. A field worker will administer questionnaires by interview with the aid of an electronic tablet, preprogrammed with prompts and validation rules to help limit missing data and ensure data quality. All field workers will be educated at the degree level in a related field (eg, health sciences) and will attend a minimum of five days of training covering all elements of the primary data collection, including a dedicated session on administering dietary recalls facilitated by a nutrition researcher. Following the training, field workers will have access to bespoke training resources.

#### Focus Groups

At baseline and follow-up, focus groups will be oriented around understanding what influences people’s food practices and exploring the role of the hypermarket within the local foodscape. We will explore the day-to-day experience of food, including travel to procure food and the influence of family and community members. We will also investigate the connection people make between food, health and well-being, and the environment.

The focus groups will be held at local venues, such as community church halls or appropriate business outlets; will be conducted by two facilitators; and will last approximately two hours. Focus groups will be held for male and female participants as well as for lower- and higher-SES participants separately. Each focus group will start with a short introduction to the project, described as research on the food local people eat and how they get it. Using a topic guide, the facilitator will help to guide and explain the questions, a second facilitator will take notes, and the discussions of each focus group will be tape-recorded. The audio recordings will be transcribed verbatim and, if applicable, translated into English.

#### Stakeholder Interviews

At baseline, stakeholder interviews in the intervention area will be oriented around determining how decisions to invest in and situate the mall and hypermarket were made as well as projected impacts in relation to food prices and variety, other food outlets in the area, food supply chains, and health among the local people. At follow-up, interviews will focus on the implementation of the intervention and any important short-term outcomes. In the comparison area, stakeholder interviews at both time points will focus more broadly on the impacts of the changing local foodscape.

The interviews will be held at a location convenient to the stakeholder, likely their place of work. Semistructured interviews will be conducted by two facilitators according to a topic guide and will last for approximately one hour. The interviews will be tape-recorded; the audio recordings will then be transcribed verbatim and, if applicable, translated into English.

#### Foodscape Audit

At baseline and follow-up, systematic on-the-ground data collection will be undertaken to geolocate and categorize food outlets in each of the local study areas. Teams of field workers will systematically walk around the study areas, coding the location and types of establishments and taking photos of food outlets. Data collection will be undertaken using electronic tablets. In addition, we will explore the availability and accuracy of existing data, such as county records of licensed food sellers.

#### Shopper Intercept Survey

At follow-up, shoppers in the hypermarket will be intercepted while they shop and asked a set of standard questions covering basic demographics, the items being purchased on the shopping trip, the mode of transport used to get there, and the main reasons for using the hypermarket. A field worker will administer the survey by interview with the aid of an electronic tablet. It is anticipated that each survey will take 5 to 10 minutes.

#### Data Management

The study will be conducted in accordance with relevant current policies, standard operating procedures, and regulatory requirements for data protection, storage and security, and secure data sharing across sites at the Kenya Medical Research Institute and the University of Cambridge. Each participant will be assigned a unique registration number. A master list linking each participant registration number to identifying details will be stored separately from the deidentified data. There will be secure storage of both paper (ie, locked filing cabinet) and electronic (ie, password-protected database) data at the Kenya Medical Research Institute. Electronic data will be stored on a secure server at the Kenya Medical Research Institute.

#### Quantitative Data

For the household survey, foodscape audit, and shopper intercept survey, data will be captured electronically using preprogrammed forms with existing prompts, codes, and validation rules to help limit missing data and ensure data quality using the mobile app CommCare (Dimagi). Additional quality control checks will be undertaken once these quantitative data are collated in the database.

Raw survey data will be collected, stored, and backed up on restricted-access CommCare cloud servers. Raw data sets will be downloaded by a data manager onto local password-protected computers for additional backup and management. Data cleaning will be undertaken using Stata software (StataCorp), in which data will be checked for inconsistencies, variables manipulated, and new variables derived. A cleaned data set will be generated and stored locally alongside cleaning and analysis files. Cleaned, deidentified data sets will be shared between institutions according to local ethical approval and the terms of a formal data sharing agreement.

#### Qualitative Data

For the qualitative focus groups and stakeholder interviews, all transcripts will be checked for accuracy by specially trained qualitative coders prior to analysis. Transcripts will then be imported into NVivo software (QSR International) for coding.

### Analysis Methods

#### Household Survey

For all quantitative analyses, the level of statistical significance will be set as α<.05. All participants who provide data will be included in analyses, and we will not impute missing data. Net changes between baseline and follow-up will be computed, and significance tests will be conducted using a method appropriate to the distribution of the data. Differences in means will be compared using *t* tests or analyses of variance for normally distributed variables, while medians will be compared using the Wilcoxon rank-sum test or the Kruskal-Wallis test for skewed data. We will further undertake multivariable regression analyses adjusted for important covariates, using models appropriate to the distribution of the data and accounting for clustering by household. For binary outcomes, we will explore the use of logistic regression models, and for continuous outcomes we will consider linear regression models and negative binomial or zero-inflated generalized linear models. Regression estimates and 95% confidence intervals will be reported.

#### Focus Groups and Stakeholder Interviews

The qualitative focus groups and semistructured interviews will be analyzed by a team of qualitative coders using thematic analysis. Double-coding of selected transcripts will be used to facilitate this process. Researchers will develop a codebook based on *a priori* themes from the topic guides, and new codes may also emerge. All codes will be combined into a master codebook, and this will be used for analysis.

#### Foodscape Audit

Food outlets will be counted and categorized (eg, restaurant, kiosk, and supermarket) descriptively. Simple change scores will be calculated. Geospatial information will be imported into a GIS and used to inform analyses exploring distance between households and food outlets as well as density of food outlets.

#### Shopper Intercept Survey

Intercept survey data will be analyzed using descriptive statistics. This data will be collected once the hypermarket has opened.

#### Mixed Methods Analysis

To integrate the study findings, a mixed methods, sequential explanatory design will be used. This design consists of two distinct phases: quantitative followed by qualitative [29]. Quantitative data will be analyzed first and take priority in the design. Following this, qualitative data will be analyzed to help explain, or elaborate on, the quantitative results. The methods will be mixed at the analysis stage. This has similarities to the approach of following of a thread [30,31], where a question or theme taken from one research phase is carried across another phase. This proposal differs slightly from a traditional explanatory design in that the data collection will take place in parallel, but the analysis will take place sequentially.

The primary reason for mixing the methods is to offset the strengths and weaknesses of each method to provide greater insight than could be gleaned from either method alone. For example, the quantitative thread benefits from larger numbers but does not provide insight into how or why outcomes are brought about. By contrast, the qualitative data will provide complementarity via the explanation, elaboration, and illustration of the results from the quantitative method, but is limited by the smaller sample size. The two approaches are intended to answer different questions: *how* (ie, quantitative thread) and *why* (ie, qualitative thread). The qualitative thread will allow for some exploration of potential causal inference and mechanisms that would not otherwise be possible from the quantitative design. In addition, the qualitative thread will provide insight into the local context and give some indication of the potential generalizability of findings.

### Knowledge Exchange and Dissemination

#### Knowledge Exchange

It is anticipated that findings from this study will be relevant for a range of policy areas, including nutrition, health and health inequalities, economic development, urban planning, agriculture, and food supply. Findings will be directly relevant to decision makers in the local study areas. In addition, given the similarity between this development and others taking place in low- and middle-income contexts, as well as wider considerations of health and economic or urban development, there is scope for considerable generalizability of these findings.

There will be 3 key windows for knowledge exchange within the study:

1. An initial stakeholder mapping phase will be an opportunity to understand the landscape of the public and private decision makers involved in this process as well as their needs and practices in terms of evidence provision and use.
2. The stakeholder interviews will provide opportunities to further develop this understanding as well as stakeholder perspectives on health and economic development.
3. The dissemination phase will involve sharing the results with the above actors in a way that reflects our increased understanding of their perspectives. Beyond the local stakeholder, we will also engage with national, regional, and international decision makers.

Key messages for our knowledge exchange will be guided by the research findings. The methods for dissemination will be guided by what is likely to be most effective for the relevant stakeholders.

#### Dissemination

Findings will be communicated to study participants through activities such as meetings based on the principle and approaches of *Baraza*—informative or deliberative public meetings held in communities in Kenya—and other existing networks and forums that can engage widely within communities. We will communicate study findings through academic publications and conference presentations. In addition, we will pursue a number of nonacademic outputs, activities, and events, including the production of lay summaries, blogging and social media, communicating via news media, and further *Baraza*-style meetings for different community and stakeholder audiences.

### Monitoring

#### Data Monitoring and Auditing

This study forms part of the Global Diet and Activity Research Group and Network (the Network). The Network has a Steering Group comprised of senior investigators across the participating sites, which meets every 6 weeks. The Network Steering Group will act as a study steering committee, providing general oversight and advice on scientific and operational issues. Because of the low-risk nature of the study and the lack of a researcher-manipulated intervention, there will be no formal data monitoring committee for this study. There are no planned interim analyses or stopping guidelines. Minimal monitoring will be undertaken by a member of the investigative team to verify that study procedures are undertaken in accordance with the protocol by communicating deviations where they occur and taking action to prevent recurrence, auditing the completeness of consent forms, and auditing the accuracy and completeness of study data.

#### Harms

This is considered a minimal-risk study as defined by the local ethics committee. As such, a formal process of adverse event monitoring will not be implemented. However, if any member of the study team becomes aware of a serious adverse event (ie, its occurrence leads to hospitalization, death, or permanent disability or is life threatening), it will be reported to the principal investigator, who will report this to the local ethics committee according to the committee’s standard procedures.

### Ethics

Ethical approval has been received from the Kenya Medical Research Institute Scientific and Ethics Review Unit (reference KEMRI/SERU/CGHR/174/3730). Important protocol modifications will be updated in the study protocol and a new version released. Protocol amendments will be approved by the Kenya Medical Research Institute Scientific and Ethics Review Unit.

All study participants will read an information sheet and provide written informed consent prior to any study procedures taking place. Participants aged 18 years or over who cannot read and write may thumb-print their consent after the document has been read to them in the presence of their chosen witness. All participants aged 18 years or over who are able to write will sign the consent forms. Parents or guardians will sign consent forms on behalf of their children who are under 18 years of age. An assent form will also be signed by children who are under 18 years or age, or a thumbprint will be given if they cannot read or write, after information has been read to them. The consent and assent forms will be translated into Luo and Kiswahili, the local language and national language, respectively.

Data will not be copied, removed, or disclosed to anyone outside the collaborating investigators and approved study staff. Approved staff will be given password-restricted access to the data. All participants are entitled to request the details of any personal data relating to them. Confidentiality will be maintained by storing consent forms separately from deidentified data, which will use registration numbers. Deidentified data will be shared between the Network partners under the terms of a formal collaboration and data sharing agreement. Identifiable data will be stored at the Kenya Medical Research Institute only and will not be shared.

## Results

As of December 2020, we have undertaken initial stakeholder mapping and completed the baseline assessment. The follow-up assessment, data analysis, and publication are anticipated to take place in 2021.

### Initial Stakeholder Mapping

In November 2018, stakeholders were identified and a meeting convened in each of the study areas. In Kisumu (ie, the hypermarket area), the meeting was attending by 32 stakeholders, including representatives from the Ministry of Health, the Lake Basin Development Authority, the county health department, trade and agriculture departments, faith and religious leaders, community health volunteers, and the private food retail and transport industries. In Homabay (ie, the comparison area), the meeting was attended by 19 stakeholders mostly representing the county health department. Investigators briefly described the study and chaired an open forum discussion in which stakeholders could reflect on aspects relevant to them.

### Baseline Assessment

We undertook baseline assessments in Kisumu (ie, the hypermarket intervention area) in March 2019 and in Homabay (ie, the comparison area) in June 2019. From a total sampling frame of 400 households, we recruited 376 of these, giving an overall response rate of 94.0%. This exceeded our target of recruiting 250 households at baseline. Numbers of respondents completing each element of the assessment are reported in Table 1. We met or exceeded target sample sizes for all elements of the assessment.

**Table 1:** Baseline assessment conducted in 2019.

Assessment		Kisumu (ie, hypermarket area), n (%)	Homabay (ie, comparison area), n (%)
**Household survey**			
	Households (N=376)	180 (47.9)	196 (52.1)
	Individuals (N=516)	260 (50.4)	256 (49.6)
Focus groups (N=8)		4 (50)	4 (50)
Stakeholder interviews (N=44)		21 (48)	23 (52)
Foodscape audit: outlets geocoded (N=1920)		1008 (52.5)	912 (47.5)

## Discussion

### Overview

In this paper, we report the protocol for a mixed methods, natural experimental study evaluating the impacts of a new hypermarket on dietary behavior and other related outcomes among local residents in Western Kenya. In addition, we aim to develop a broader understanding of the impacts of a rapidly changing local foodscape, the meaning that local people attribute to these changes, and local people’s food practices more generally. A number of reflections on study design and conduct arising from this study are likely to be of interest to those undertaking similar work.

### Practical Challenges of Natural Experimental Designs

A defining feature of natural experimental designs is that the intervention of interest is not controlled by researchers, which can raise practical challenges for evaluation. Since the timing of the intervention is not under researcher control, it can be difficult to complete baseline assessments in the interval between the identification of the natural experiment opportunity and the implementation of the intervention. Equally, if an intervention is delayed, it can be difficult to complete an evaluation within the time constraints of research funding. In this study, the opening of the Lake Basin Mall and the hypermarket has been delayed. The baseline assessment has been completed, allowing detailed quantitative and qualitative exploration of associations between food retail, including supermarkets, and the outcomes of interest, which is an important contribution to the broader goal of understanding the role of food retail in shaping dietary and other health behaviors.

### Natural Experimental Designs in Low- and Middle-Income Contexts

To date, much of the evidence from research on supermarkets, and studies using natural experimental designs, is drawn from high-income countries. However, the evidence generated from these types of studies tends to be at least somewhat specific to the context, and it is unlikely that findings from high-income countries would be completely generalizable to LMICs. In particular, key differences in LMICs include resource constraints from the individual to the governmental level, the co-occurring burden of infectious disease, and, particularly for research on diet, the nutrition transition and triple burden of malnutrition. It is likely that the identification of generalizable causal relationships from natural experimental studies will rely on the cumulation of studies across different contexts [20,32]. It is, therefore, important that evidence from a range of different contexts, including LMICs, forms part of this corpus of work.

### Conceptualizing Exposure in Natural Experimental Designs

With natural experimental designs, the delineation of exposed (ie, intervention) and unexposed (ie, comparison) groups is required in order to take advantage of the variation in exposure generated. This is not always straightforward and depends on the research question and putative causal chain. For example, the use of area-based exposures is often used [33], encompassing individuals within a defined geographical area. Graded individual-level exposures can also be considered, commonly operationalized as distance to the intervention of interest. In this study, we operationalized a group-level exposure as living in either the hypermarket intervention area (ie, Kisumu) or comparison area (ie, Homabay). The group-level comparison was intended to capture both direct and indirect impacts of the hypermarket, independent of whether the individual actually shopped there, possibly via associated changes to local food retail. We also used an individual-level exposure, operationalized according to whether the individual shopped at the new hypermarket. This was intended to allow for the detailed evaluation of direct impacts among shoppers.

The use of controlled comparisons is intended to account for confounders. However, it is acknowledged that comparison groups in natural experimental studies are typically imperfect and not akin to those achieved through randomization. Groups that are broadly similar across a range of important demographic, environmental, and behavioral variables are often the best that can be achieved. In this study, we acknowledge that our comparison area differed in some respects to the intervention area; nevertheless, this design will still allow researchers to account for measured confounding. The possibility of unmeasured confounding related to other concurrent changes is a core limitation of this study and of natural experimental designs in general.

### Strengths and Limitations

This is one of very few studies examining how changes in the food environment relate to changes in behavior and health in LMICs. The strengths of the study include the use of established and validated quantitative methods and the collection of complementary qualitative data, making this a unique data set for this setting. We particularly focus on inequality and the potential for differing impacts in different SES groups. The limitations of this study relate mainly to the general limitations of natural experimental designs and are discussed in detail in the previous section.

### Conclusions

This study aims to further the understanding of the relationship between food retail and dietary behaviors in Kenya. Baseline assessments for the study have been completed.

## Abbreviations

GIS: geographic information system
LMICs: low- and middle-income countries
NIHR: National Institute for Health Research
SES: socioeconomic status
The Network: Global Diet and Activity Research Group and Network
